# 

**Published:** 1882-08

**Authors:** 


### ARTICLE IX.

THE TRI-STATE MEDICAL SOCIETY.—This flourishing association promises well for its next meeting. Sixty papers have been promised, and are now catalogued for publication. As each is limited to twenty-five minutes, and some will not exceed ten minutes in length, there will be time for all, for this society holds three working sessions each day, and has no time for banquets, receptions, etc.

The meeting for 1882 will be in Terre Haute, September 26, 27 and 28, and the railroads and hotels have promised to contribute to its success.

The rapid growth, and the good work done by this society, have stimulated the workers in the profession of Indiana, Kentucky and Illlinois, and in Cincinnati and St. Louis, to renewed effort. Hence its rapid growth.

Dr. T. M. Holloway, of Louisville, is President, and Dr. G. W. Burton, of Mitchell, Ind., Secretary.

The programme will be ready for notice in our September issue.

---

### HERPES NIGER OF THE LABIA MAJORA.

The woman, twenty-one years of age, was at the end of her gestation. There was a gangrenous patch on the lower part of the abdomen, eight cm. long and six cm. broad, near the pubis. After parturition a high fever set in, the lochia being normal; pulse 140; no vomiting. On the right labium there appeared some spots of herpes, which extended all over the pubes in the next three days, the fever increasing at the same time. The vesicles contained a dark-colored fluid, and they were isolated. The gangrenous patch began to heal, but the herpes spread to the perineum. The patient died after six days.—*L' Union Médicale.*

CLINICS.

MONDAY.

Eye and Ear Infirmary—1.15 p. m., Otological, by Prof. Jones; 2.15 p. m., Ophthalmological, by Prof. Hotz.

Mercy Hospital—2 p. m., Medical, Profs. Hollister and Quine.

Rush Medical College—3 p. m., Dermatological and Venereal, by Prof. Hyde.

Woman's Medical College—2 p. m., Dermatological and Venereal, by Prof. Maynard.

TUESDAY.

Cook Co. Hospital—2 to 4 p. m., Medical and Surgical Clinics.

Mercy Hospital—2 p. m., Surgical Clinic, by Prof. Andrews.

Woman's Medical College—10 a. m., Prof. Ingals.

WEDNESDAY.

Chicago Medical College—2 p. m., Eye and Ear, by Prof. Jones.

Rush Medical College—2 p. m., Medical, by Prof. Bridge; 3 p. m., Ophthalmological and Otological, by Prof. Holmes; 3:30 to 4:30 p. m., Diseases of the Chest, by Dr. E. Fletcher Ingals.

Woman's Medical College—2 p. m., Eye and Ear, by Dr. W. T. Montgomery; 3 p. m., Diseases of Children, by Prof. Chas. W. Earle.

Eye and Ear Infirmary—2.30 p. m., Dr. E. J. Gardiner.

THURSDAY.

Chicago Medical College—2 p.m., Gynæcological, Prof. Dudley.

Rush Medical College—2 p. m., Diseases of Children, by Prof. Knox; 3 p. m., Diseases of the Nervous System, by Prof. Lyman.

Eye and Ear Infirmary—2 p. m., Ophthalmological, by Dr. Hotz.

Woman's Medical College—3 p. m., Surgical, by Prof. Owens.

FRIDAY.

Cook County Hospital—2 to 4 p. m., Medical and Surgical Clinics, by Profs. J. H. Hollister and R. N. Isham.

Mercy Hospital—2 p. m., Medical, by Prof. Davis.

SATURDAY.

Rush Medical College—2 p. m., Surgical, by Prof. Gunn.

Mercy Hospital—2 p. m., Surgical Clinic, by Prof. Andrews.

Chicago Medical College—3 p. m., Neurological, Prof. Jewell.

Woman's Medical College—2 p. m., Gynæcological, by Prof. Stevenson.

Daily Clinics, from 2 to 4 p. m., at the Central Free Dispensary, and at the South Side Dispensary.

